# RelVid: Relational Learning with Vision-Language Models for Weakly Video Anomaly Detection

**DOI:** 10.3390/s25072037

**Published:** 2025-03-25

**Authors:** Jingxin Wang, Guohan Li, Jiaqi Liu, Zhengyi Xu, Xinrong Chen, Jianming Wei

**Affiliations:** 1 Shanghai Advanced Research Institute, Chinese Academy of Sciences, Shanghai 201210, China; wangjx2022@shanghaitech.edu.cn (J.W.); ligh@sari.ac.cn (G.L.); liujiaqi2024@sari.ac.cn (J.L.); wjm@sari.ac.cn (J.W.); 2 School of Information Science and Technology, ShanghaiTech University, Shanghai 201210, China; 3 School of Electronic, Electrical and Communication Engineering, University of Chinese Academy of Sciences, Beijing 100049, China; 4 Academy for Engineering and Technology, Fudan University, Shanghai 200433, China

**Keywords:** vision-language model, Adapter, weakly video anomaly detection, feature learning

## Abstract

Weakly supervised video anomaly detection aims to identify abnormal events in video sequences without requiring frame-level supervision, which is a challenging task in computer vision. Traditional methods typically rely on low-level visual features with weak supervision from a single backbone branch, which often struggles to capture the distinctive characteristics of different categories. This limitation reduces their adaptability to real-world scenarios. In real-world situations, the boundary between normal and abnormal events is often unclear and context-dependent. For example, running on a track may be considered normal, but running on a busy road could be deemed abnormal. To address these challenges, RelVid is introduced as a novel framework that improves anomaly detection by expanding the relative feature gap between classes extracted from a single backbone branch. The key innovation of RelVid lies in the integration of auxiliary tasks, which guide the model to learn more discriminative features, significantly boosting the model’s performance. These auxiliary tasks—including text-based anomaly detection and feature reconstruction learning—act as additional supervision, helping the model capture subtle differences and anomalies that are often difficult to detect in weakly supervised settings. In addition, RelVid incorporates two other components, which include class activation feature learning for improved feature discrimination and a temporal attention module for capturing sequential dependencies. This approach enhances the model’s robustness and accuracy, enabling it to better handle complex and ambiguous scenarios. Evaluations on two widely used benchmark datasets, UCF-Crime and XD-Violence, demonstrate the effectiveness of RelVid. Compared to state-of-the-art methods, RelVid achieves superior performance in both detection accuracy and robustness.

## 1. Introduction

In real-world applications, anomalies are not only widespread but also potentially harmful, which makes anomaly detection a critical challenge. The diverse nature of anomalies, coupled with their infrequent occurrence, complicates the acquisition of sufficient labeled data for training robust models. This has fostered an increasing interest in weakly supervised video anomaly detection (WVAD). Unlike semi-supervised methods that do not require labels, WVAD relies on a small number of video-level labels to provide more accurate and reliable anomaly detection results.

**Figure 1:**
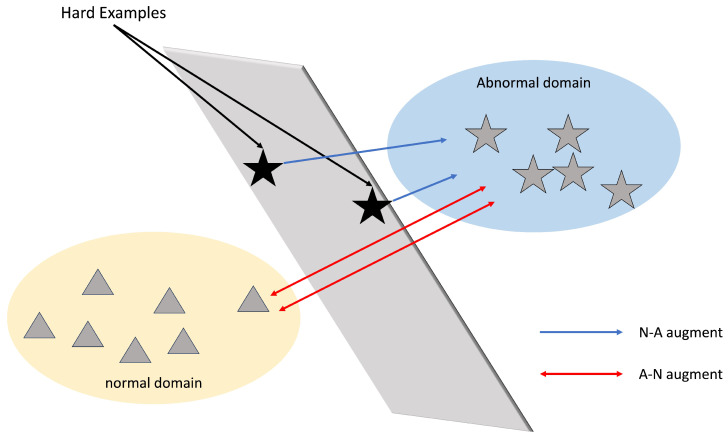
Illustration of the core feature adjustment process in WVAD: “Hard examples” are data points that are difficult to classify correctly and typically lie on the boundary between the normal and anomaly domains, contributing to reduced accuracy.

Traditionally, the anomaly detection pipeline involves dividing a video into multiple segments, extracting global features from each segment using pre-trained vision models, and then applying multi-instance learning (MIL) followed by binary classification, as demonstrated in Figure 1. With the evolution of deep learning models, vision models have advanced from earlier approaches such as C3D [1,2,3] and I3D [4,5,6,7,8], In addition to RGB information, inspired by action recognition [9], some studies [10,11] also utilize optical flow features. Subsequently, many have continued to adopt MIL (multiple-instance learning) methods, with some focusing on feature aggregation to enhance the model’s capability. For instance, RTFM [12] uses top-k scores instead of the highest score to improve score prediction. UMIL [13] learns unbiased anomaly features, which contribute to the improvement of WSVAD by mitigating the effects of contextual variations. However, relying solely on image information poses significant limitations, as it heavily depends on the quality and quantity of available images, which can restrict the model’s overall performance and generalization ability. Specifically, in the case of videos, in addition to image data, there are other valuable sources of information, such as audio and text. Consequently, leveraging diverse types of information to detect anomalies has become a recent focus of research in the academic community, and integrating different types of image information is a commonly used approach [10]. With the introduction of larger datasets like XD-Violence [5], which includes more diverse information such as audio, there has been a shift toward utilizing multiple data modalities. Some studies, such as by Fan et al. [14], used both image and audio information by employing VGGish to process audio data and concatenating it with image features for video anomaly detection. Wu et al. introduced AVVD [15], a method that enhances anomaly detection by integrating image, optical flow, and audio data through a three-branch neural network architecture. This approach improves cross-modal anomaly detection by leveraging the complementary information from these diverse data sources. However, it faces challenges in effectively modeling the relationships between these modalities, which limits its efficiency and robustness. While features from diverse modalities can provide valuable information, they also risk introducing more noise and necessitate complex structures to balance effectively. Therefore, some studies, such as [16,17], adopted a two-stage training process utilizing pseudo-labels. In this approach, a pre-trained model is refined through a self-learning mechanism, which adaptively selects high-confidence abnormal regions as pseudo-labeled data to enhance anomaly detection. However, since the labels are derived from model predictions, their authenticity remains uncertain, which could affect the model’s reliability. Additionally, the need for two rounds of training significantly increases the overall training time, posing a potential drawback in terms of computational efficiency.

Building on the above considerations, in this paper, RelVid is introduced as a novel framework that employs a dual-branch structure to separately extract and integrate visual and textual features, thereby enhancing the model’s ability to detect anomalies more effectively. Since feature extraction is typically trained on image datasets, it is not well-suited to capture temporal dependencies in videos. Adapters are efficient components that assist the feature extractor in acquiring useful information, enabling the model to capture long-range temporal relationships from multiple perspectives. Temporal dynamics are crucial for anomaly detection, as abnormal events often manifest through subtle variations over time. Moreover, the video anomaly recognition auxiliary tasks further optimize the alignment of visual and textual features under weak supervision, preserving pre-trained knowledge while enhancing the model’s generalization ability, particularly in scenarios with limited labeled data. Several auxiliary tasks, including anomaly recognition and reconstruction, are incorporated to improve feature extraction and assist the model in distinguishing between normal and anomalous events.

The key contributions of this paper are as follows:

1. To incorporate additional modalities of information to guide model training, a new framework is proposed, consisting of three key components: the class activation module to improve feature discrimination, the Adapter for capturing temporal dependencies, and two auxiliary tasks that adapt the pre-trained model for weakly supervised anomaly detection;
2. To ensure that the features obtained from the fine-tuned feature extractor are more suitable for the video anomaly detection task, the Adapter is proposed to learn class-specific features and capture the temporal dependencies of different video events more flexibly and accurately.
3. To enhance the model’s ability to capture diverse information, auxiliary tasks that leverage both textual and visual patterns are proposed, while reconstruction tasks further support feature learning, improving the model’s overall performance.
4. The effectiveness of RelVid is demonstrated on two widely used benchmarks, UCF-Crime and XD-Violence, where it achieves state-of-the-art performance with unprecedented results: 87.71% AUC and 80.76% AP, surpassing most previous methods by a significant margin.

This paper is structured as follows: Section 2 reviews related work; Section 3 defines the problem and presents the RelVid framework; Section 4 details the experimental setup, results, and discussion; and Section 5 concludes with a summary and directions for future work.

## 2. Related Work

The video anomaly detection (VAD) task has garnered significant attention, resulting in various approaches based on different supervision modes. Among these, weakly supervised methods have gained prominence, offering substantial potential in this field.

### 2.1. Weakly-Supervised Video Anomaly Detection

Compared to semi-supervised VAD methods [6,18,19,20,21], weakly supervised VAD benefits from the availability of video-level labels, which improves model accuracy and provides clearer guidance during training. Early approaches, such as DeepMIL [1], laid the foundation for this methodology. Over time, multiple-instance learning (MIL) has become the standard technique for weakly supervised video anomaly detection (WVAD). The primary objective of WVAD is to maximize the score gap between normal and abnormal events. Score distance learning methods [10,22] aim to minimize intra-class variation and enhance inter-class separation by focusing on the highest and lowest anomaly scores. While these methods have demonstrated strong performance, MIL-based approaches face inherent limitations, particularly their reliance on high-scoring segments and regression outputs, which do not incorporate feature-based decision-making.

To address these limitations, Tian et al. [12] proposed amplifying the feature magnitude differences between classes, while Wu et al. [23] improved feature distances using center features. Later, reconstruction methods [24,25,26] from semi-supervised learning were adopted to obtain class-specific features and improve class separation. Further advancements have incorporated temporal relationships through self-attention models and Transformers [27], which capture more complex temporal dynamics and make the model more sensitive to semantic and structural information in a video context. For example, Zhong et al. [11] introduced a graph convolutional network-based approach to model feature similarity and temporal consistency across video clips. Despite these advances, the ability to discriminate difficult examples through methods like feature enhancement remains limited, highlighting the need for more robust solutions.

### 2.2. Vision-Language Learning in VAD

Early VAD methods mainly relied on visual data for feature extraction using pre-trained models like C3D [1] and I3D [4], focusing exclusively on RGB frames. However, these models overlooked the textual information that could further enhance anomaly detection. CLIP [28] revolutionized these approaches by mapping both textual and visual data into a shared latent space using similarity-based learning, enabling effective cross-modal interaction. CLIP set new benchmarks across vision-language tasks and showed great promise in downstream applications like image classification and object detection [29]. Recent efforts to incorporate CLIP into VAD have mostly focused on feature extraction, neglecting the potential of combining text and visual information for more robust anomaly detection.

Based on this, CLIP has been extended to video analysis, providing new avenues for VAD. Traditional video anomaly detection methods primarily focus on visual features, but these approaches often struggle with providing meaningful labels or explanations for detected anomalies. To address this gap and improve anomaly categorization interpretability, textual features alongside visual data are integrated. For example, VideoCLIP [30] used temporally overlapping video–text pairs to align video and text representations, enhancing contrastive learning with hard negatives. Building on this, Zanella et al. [31] integrated textual features into their models, applying multiple-instance learning (MIL) strategy for improved anomaly classification. While parts of CLIP’s capabilities have been utilized, its potential for both feature extraction and detailed anomaly labeling was not fully explored, which is addressed in this work.

## 3. Framework

The generalized framework follows the multiple-instance learning (MIL) models, where a positive bag signifies an anomaly and a negative bag represents normal instances. The components that comprise this framework are detailed in the subsequent subsections.

### 3.1. Overview

In WVAD, both normal and abnormal videos are assigned a video-level label, Y={0,1}. The dataset is represented as

(1) $$D={\left\{\left(x^{i},0\right)\right\}}_{i=1}^{M}\cup {\left\{\left(x^{j},1\right)\right\}}_{j=1}^{N},$$

where N≪M and y∈{0,1}. Here, $x^{i}$ and $x^{j}$ denote the feature representations of individual video snippets, with y=1 indicating that at least one frame within the video snippet contains an anomalous event. The goal of WVAD is to utilize these coarse video-level labels to predict anomaly scores for each individual frame within the dataset.

**Figure 2:**
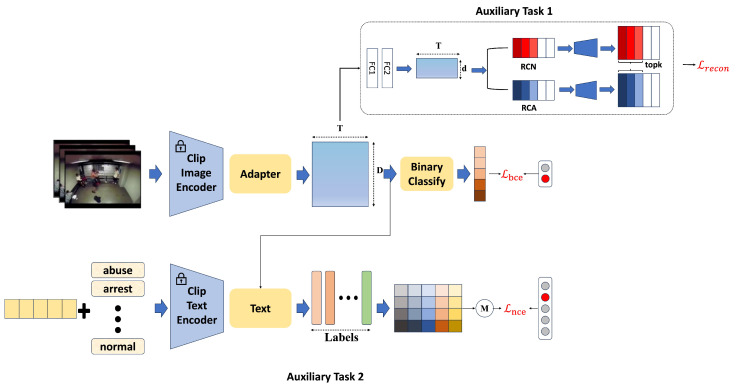
The framework of RelVid.

RelVid, as shown in Figure 2, adopts a dual-branch structure. The upper branch processes normal visual data, while the lower branch handles textual information. The entire structure consists of three main components: feature extraction, the Adapter component, and auxiliary tasks. The feature extraction component leverages a pre-trained model to extract both visual and textual features from the video and text. The Adapter component is responsible for enhancing temporal modeling, enabling the model to capture long-range dependencies and better distinguish between normal and anomalous events. Lastly, the auxiliary tasks help in fine-tuning the model by applying additional learning strategies, such as category-specific feature activation and alignment between visual and textual cues, thus improving the model’s overall performance in weakly supervised settings.

### 3.2. Feature Extraction

**Figure 3:**
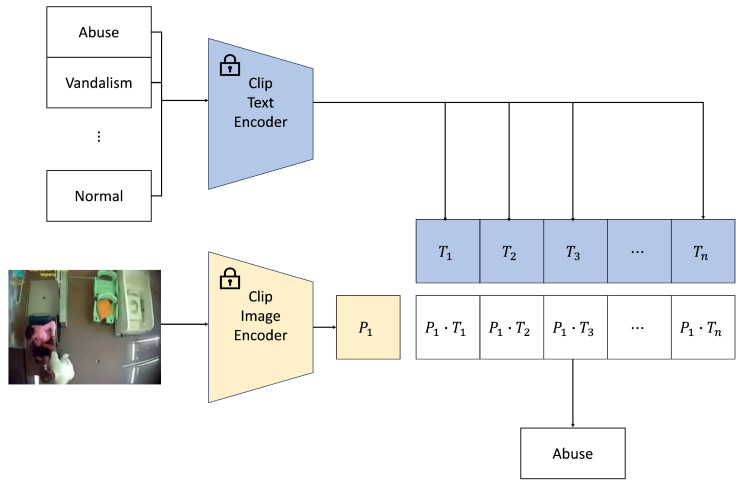
CLIP model architecture at the inference stage: alignment of modalities into a joint embedding space via contrastive learning.

Compared to traditional feature extractors, the CLIP [28] model excels at linking vision and language, mapping features from different modalities into a unified space, as illustrated in Figure 3. By using CLIP as a single pre-trained model, the challenges associated with employing multiple models for extracting information from different modalities and balancing them are effectively mitigated. This approach allows the model to leverage cross-modal knowledge, enhancing anomaly detection. This is particularly valuable in weakly supervised settings, where explicit labels are scarce, and multimodal data can provide additional context, leading to more accurate predictions. This can be formally represented as the following contrastive learning objective:

(2) $$L_{\mathrm{contrastive}}=-\log\frac{\exp(\mathrm{sim}\left(f_{\mathrm{image}}\left(x^{i}\right),f_{\mathrm{text}}\left(t^{i}\right)\right))}{\sum_{j=1}^{N}\exp\left(\mathrm{sim}\left(f_{\mathrm{image}}\left(x^{i}\right),f_{\mathrm{text}}\left(t^{j}\right)\right)\right)}$$

where sim(a,b) is the similarity measure between the image feature *a* and the text feature *b*, and $f_{\mathrm{image}}\left(x^{i}\right)$ and $f_{\mathrm{text}}\left(t^{i}\right)$ are the feature representations of the *i*-th image and its paired text, respectively. This objective ensures that matching pairs (image-text) are closer in the embedding space than mismatched pairs.

CLIP employs a Transformer-based architecture to jointly process images and text. A Vision Transformer (ViT) is used for image encoding, and a Transformer is employed for text encoding. The model is trained by optimizing the contrastive objective to align the image and text modalities. During the training process, the image and text encoders learn to map images and texts into a shared embedding space, allowing CLIP to generalize across various domains and tasks.

In the context of this framework, CLIP serves as a powerful feature extractor, capturing both visual features and the semantic relationships between images and text. The multimodal learning capability offered by CLIP significantly enhances the performance of anomaly detection tasks, where textual descriptions can provide valuable context for interpreting video sequences.

### 3.3. Adapter

#### 3.3.1. Feature Activate Module

To identify abnormal events in video segments with weak supervision, it is crucial to learn discriminative features that distinguish normal and abnormal classes. While previous methods extract features using a backbone pre-trained on large-scale action recognition datasets, these features often lack the capacity to effectively differentiate between classes. Additionally, a single backbone struggles to model inter-class variations, making class-specific processing difficult. To address these limitations, we propose an implicit class activation module inspired by [32], which uses domain-specific activation weight differentiation in cross-domain face recognition. This module enables adaptive class-specific feature differentiation, improving the model’s ability to detect anomalies in weakly labeled video data without requiring explicit architectural modifications.

**Figure 4:**
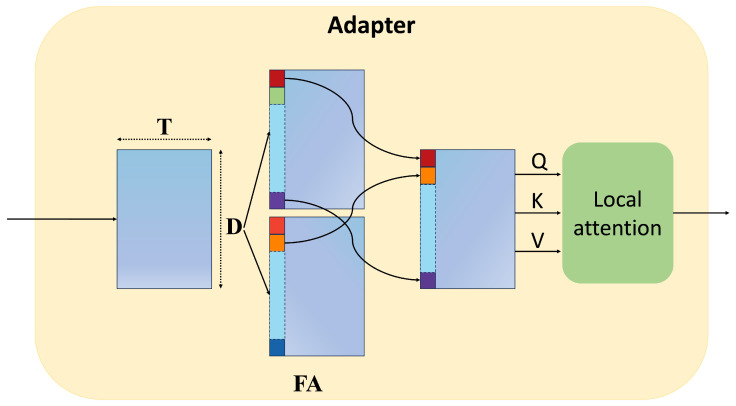
The structure of the Adapter module.

The *T* segments passed through the backbone produce *D*-dimensional features $B\in R^{T\times D}$, which are then input to the FA (Feature Activation) module running in time order. As shown in Equation (3), the channel size of *B* is expanded $\hat{B}\in R^{T\times 2D}$ according to the number of categories, where $f^{FA}$ is the feature activation (FA) module with parameters ϕ, and $\hat{B}=\left\{{\hat{b}}_{1},{\hat{b}}_{2},\ldots ,{\hat{b}}_{T}\right\}$. Then, in Equation (4), each feature vector ${\hat{b}}_{i}$ is split into ${\hat{b}}_{1}^{i}$ and ${\hat{b}}_{2}^{i}$ to achieve maximum activation. The specific frameworks are shown in Figure 4.

(3) $$\hat{B}=f_{\phi_{1}}^{FA}\left(B\right)\;B\in R^{2\times T\times D}$$

(4) $${\hat{b1}}_{i}=B\left[0\right],\;{\hat{b2}}_{i}=B\left[1\right]$$

(5) $$f_{i}=f_{\phi_{2}}\left(\max\left[{\hat{b1}}_{i},{\hat{b2}}_{i}\right]\right)+B,\;\left(i=1,2,\cdots ,T\right)$$

Through the max operation in Equation (3), class-representative information is implicitly aggregated from the backbone features *B*. Only the weight of the activated element is propagated to the gradient $\frac{𝜕f_{i}}{𝜕{\hat{b1}}_{di}}$ when ${\hat{b1}}_{di}\geq {\hat{b2}}_{di}$, and $\frac{𝜕f_{i}}{𝜕{\hat{b2}}_{di}}$ otherwise. This activation, customized to the specific characteristics of each class, has a notable impact on the simple configuration of a single Conv1D layer.

#### 3.3.2. Local Attention

To model local temporal dependencies in video sequences, we introduce a window-based Transformer encoder layer following the extraction of frame-level features $f_{i}\in R^{T\times D}$ from CLIP’s frozen image encoder, where *T* represents the video length and *D* denotes the feature dimension. While the feature activation process enhances discriminative power, it inadvertently fragments temporal continuity by suppressing inter-frame relationships. To address this limitation, we implement a local self-attention mechanism within overlapping temporal windows—an approach inspired by the localized receptive fields of convolutional operations.

Specifically, the temporal dimension of frame-level features is partitioned into fixed-length windows with overlapping segments. Within each window, self-attention computations are confined to establish local temporal correlations, while the explicit information exchange between adjacent windows is intentionally restricted. This design achieves dual objectives: it preserves the positional sensitivity of convolutional architectures through constrained receptive fields, effectively capturing short-range temporal patterns; and the window-based computation reduces the quadratic complexity of standard global self-attention from $O(T^{2})$ to O(T×W), where *W* denotes the window size, enabling efficient processing of long video sequences. The overlapping window strategy further mitigates potential boundary effects while maintaining temporal coherence across adjacent segments.

### 3.4. Auxiliary Tasks

**Reconstruction task:** The feature $F(F_{n},F_{a})$, implicitly activated by the FA module, is passed through the fully connected (FC) layers to predict the anomaly score $S(S_{n},S_{a}\in R^{T})$. In the feature extraction process, a reconstruction loss function $L_{recon}$ is introduced to align the features of each class in a consistent manner. The reconstruction-based approach, commonly used in semi-supervised VAD [24,25,26], reconstructs training data consisting solely of the normal class through an encoding-decoding process. The model learns the underlying data patterns or distributions by minimizing the difference between the input and the reconstructed output.

Building on this method, as shown in Figure 2, the reconstruction modules, RCN and RCA, are introduced to reconstruct the *D*-dimensional feature *F* (comprising $F_{n}$ and $F_{a}$), where $F_{n}$ and $F_{a}$ represent the normal and abnormal input snippets, respectively. This is done through two fully connected (FC) layers. The resulting features are then passed into their respective reconstruction modules for normal and abnormal instances, ensuring that each module processes the corresponding feature set effectively.

(6) $${\left\{F_{n}^{FC2_{i}}\right\}}_{i=\mathrm{topk}}\;\mathrm{for}\;\mathrm{RCN}\;\mathrm{and}\;{\left\{F_{a}^{FC2_{i}}\right\}}_{i=\mathrm{topk}}\;\mathrm{for}\;\mathrm{RCA},$$

which are then used by each module for reconstruction into a single class feature as $F_{n}^{\prime \mathrm{topk}}$ and $F_{a}^{\prime \mathrm{topk}}$, respectively. In Equation (7), the reconstruction loss $L_{recon}$ utilizes an L1 loss to minimize the discrepancy between the predicted output features and the class-specific discriminative features $F_{n}^{\mathrm{topk}}={\left\{F_{n}^{i}\right\}}_{i=\mathrm{topk}}$ and $F_{a}^{\mathrm{topk}}={\left\{F_{a}^{i}\right\}}_{i=\mathrm{topk}}$, thereby ensuring that each class feature captures the relevant representative information. These reconstruction modules with $L_{recon}$ act as auxiliary branches that are discarded during the test phase.

(7) $$L_{recon}=L_{1}\left(F_{n}^{\mathrm{topk}},F_{n}^{\prime \mathrm{topk}}\right)+L_{1}\left(F_{a}^{\mathrm{topk}},F_{a}^{\prime \mathrm{topk}}\right)$$

The use of L1 loss in the reconstruction loss function $L_{recon}$ minimizes the discrepancy between predicted and class-specific features while promoting sparsity, helping the model focus on the most discriminative features for anomaly detection. L1 loss is also more robust to outliers than L2 loss, making it suitable for handling noisy or anomalous samples in video anomaly detection, ultimately improving the model’s ability to distinguish between normal and abnormal features during training.

**Video Anomaly Recognition Task** Accurately constructing textual prompts that describe various video event categories is essential for aligning text with corresponding video frames. However, writing detailed textual descriptions for a wide range of videos is impractical. To address this challenge, a structured approach leveraging anomaly labels as a dictionary can be employed, wherein each label is systematically matched to every image to maximize textual alignment with diverse visual content. Inspired by CoOp [33], deep learning techniques can be employed to learn adaptive prefixes that align more effectively with a broader set of video frames. This approach enables the generation of more flexible and efficient textual prompts, improving the alignment between text and video content.

A learnable template can be represented as follows:

(8) $$l_{text}=\left(\epsilon_{1},\epsilon_{2},\cdots ,\epsilon_{l},\mathrm{Tokenizer}\left(\mathrm{label}\right)\right)$$

where $\epsilon_{i}$ represents the *i*th learnable vector. The Tokenizer function converts all labels, such as `fighting’, `vandalism’, or `road accident’, into vector representations. Additionally, to enhance the informativeness of $l_{text}$, location information is incorporated. The following method illustrates this approach:

(9) $$F_{text}=\xi_{text}\left(\mathrm{position}⊕l_{text}\right)$$

This integration ensures that $l_{text}$ is enriched with more relevant information, facilitating a better alignment between textual and visual representations.

By leveraging the image and text matching capability, a multiclass alignment score can be obtained. This score can then be used to guide the training of our model. The MIL-Align mechanism is proposed, which is similar to vanilla MIL. Specifically, an alignment map *M* is considered, which expresses the similarity between frame-level video features and all class embeddings. For each row, the top *K* similarities are selected, and the average is computed to measure the alignment degree between the video and the current class. A vector $S=\{s_{1},\cdots ,s_{m}\}$ is then obtained, representing the similarity between the video and all classes. The goal is for the video and its paired textual label to emit the highest similarity score among all others.

To achieve this, the multi-class prediction is computed as follows:

(10) $$p_{i}=\frac{\exp(s_{i}/\tau )}{\sum_{j}\exp\left(s_{j}/\tau \right)}$$

where $p_{i}$ is the prediction with respect to the *i*-th class, and τ refers to the temperature hyperparameter for scaling.

### 3.5. Objective Function

The top-K mechanism, as utilized in previous works [1,12], is employed to select the K highest anomaly confidences from both abnormal and normal videos as the video-level predictions. Subsequently, binary cross-entropy is applied between the video-level predictions to compute the classification loss $L_{\mathrm{bce}}$.

Overall, the final total objective of RelVid is given by the following:

(11) $$L=L_{\mathrm{bce}}+L_{\mathrm{nce}}+L_{\mathrm{recon}}$$

## 4. Experiment

### 4.1. Datasets

The proposed model was evaluated on the following benchmark datasets:

***UCF-Crime Dataset:*** UCF-Crime is a large-scale real-world dataset designed for WVAD. It spans a total of 128 h and includes 1900 surveillance videos, covering 13 anomaly event categories. Of these, 1610 videos with video-level labels are used for training, while 290 videos with frame-level labels are reserved for testing.

***XD-Violence:*** XD-Violence is another extensive dataset for violence detection, collected from a variety of sources, including movies, online videos, surveillance footage, and CCTV systems. With a total duration of 217 h, it contains 4754 videos across 6 anomaly event categories. Of these, 3954 videos are designated for training, with the remaining for testing.

### 4.2. Evaluation Metric

For each dataset, the evaluation metrics recommended by the dataset authors are followed.

For the UCF-Crime dataset, performance is evaluated using the area under the curve (AUC) for both the frame-level receiver operating characteristic (ROC) and anomalous videos (Ano-AUC), as outlined in [1]. Ano-AUC specifically assesses the model’s ability to detect rare anomalous events, which is particularly important in the presence of class imbalance.

For the XD-Violence dataset, due to significant class imbalance, the AUC metric tends to yield overly optimistic results. Therefore, we use the precision–recall curve (PRC) and average precision (AP), which focus on the positive class (violent events), to provide a more accurate evaluation, following the recommendations in [5].

For fine-grained WSVAD, we adhere to the standard evaluation protocol in video action detection, computing the mean average precision (mAP) at different intersection-over-union (IoU) thresholds ranging from 0.1 to 0.5 with a step size of 0.1. The average mAP (AVG) is also reported, with mAP values computed only for anomalous videos in the test set.

### 4.3. Implementation Details

In this work, the network structure utilizes a pre-trained CLIP (ViT-B/16) model for both image and text encoders. The image encoder is kept frozen, while only the final projection layer of the text encoder is unfrozen for fine-tuning. Both encoders share a feature dimension of 512, ensuring consistent representation across modalities. The architecture incorporates a standard feedforward network (FFN) layer from the Transformer, with GELU activation replacing ReLU. The hyperparameters are set with τ as 0.07 in Equation (10), and a context length *l* of 20. In the Adapter, the window length is set to 64 for XD-Violence and 8 for UCF-Crime. RelVid is trained on a single NVIDIA RTX 3090 GPU using PyTorch 2.1.2, with an AdamW optimizer and a batch size of 64. The learning rate is set to $2\times 10^{-5}$ and the total epochs are 20 for XD-Violence, while for UCF-Crime, the learning rate is set to $1\times 10^{-5}$ and the total epochs are 10. The experimental setup consists of an NVIDIA RTX 3090 GPU with 24 GB memory, an Intel Xeon Platinum 8362 CPU with 28 virtual cores, and 90 GB of RAM, maintaining consistency across all experiments.

### 4.4. Comparison with State of Art

The comparison of the proposed method with other state-of-the-art methods, including the semi-supervision method [18,19] and weakly-supervision methods [1,9,12,14,15,16], on the UCF-Crime dataset is detailed. The results are presented in Table 1 and Table 2. The methods are evaluated based on the area under the curve (AUC) and anomaly AUC (Ano-AUC) for the UCF-Crime dataset and average precision (AP) for the XD-Violence dataset. Additionally, we include a comparison using the fine-grained MAP@IOU metric. The experiments are divided into two parts: the first focuses on coarse categorization, while the second addresses fine categorization.

**Coarse-grained WVAD Results:** Based on the comparison results of the UCF-Crime dataset presented in Table 1, it is clear that the top three semi-supervised models, which use the same extracted features as our model, perform simple binary classification. However, all of them exhibit AUC scores around 50%, indicating that the extracted features alone do not contain sufficient distinctive information. This suggests that the features lack both temporal coherence and inter-class discriminative power. The absence of meaningful feature relationships and class separability underscores the importance of feature refinement and class-specific modeling, which our approach addresses through fine-tuned feature extraction and enhanced feature disentanglement. Later, most methods employing self-supervision improve their scores to near 80%. However, they still lag significantly behind the WVAD method, which demonstrates that textual guidance is crucial for achieving superior performance.

Among the weakly supervised learning methods we evaluated, RelVid demonstrated a significant performance improvement. Compared to other methods, RelVid performed exceptionally well in both AUC and Ano-AUC metrics. Specifically, the method by Sultani et al. [1] achieved an AUC of 84.14% and an Ano-AUC of 63.29%; RTFM achieved an AUC of 85.66% and an Ano-AUC of 63.86%; and AVVD achieved an AUC of 82.45% and an Ano-AUC of 60.27%. More importantly, among all comparison methods, AVVD used fine-grained class labels, but it did not obtain a higher score. It shows that the risk coexists with the introduction of different modal features. Therefore, it is crucial to employ other strategies to balance these diverse modal features. Ju et al. [9] achieved an AUC of 84.72% and an Ano-AUC of 62.60%; UMIL achieved an AUC of 86.75%, but did not report Ano-AUC; Zhang et al. [16] and Fan et al. [14] achieved AUC values of 86.22% and 86.19%, respectively, with no Ano-AUC values provided. Fan et al. [14] relied solely on an attention mechanism, and by placing greater emphasis on anomalies, the model’s performance was significantly enhanced, resulting in a satisfactory score. RelVid achieved an AUC of 87.71% and an Ano-AUC of 69.29%. These results demonstrate that RelVid outperforms all the compared methods in both AUC and Ano-AUC, with particularly notable improvement in Ano-AUC, highlighting its superiority in weakly supervised learning tasks.

To further evaluate the proposed method, an experiment was also conducted on the XD-Violence dataset, and the results are presented in Table 2. The evaluation for this dataset is conducted using the average precision (AP) metric, as recommended by the dataset authors [5].

For semi-supervised methods, the results further demonstrate that the extracted features provide limited useful information for the model in identifying anomalous events. Additionally, most semi-supervised models focus on small and relatively simple datasets. Due to the training modes requiring significant time and computational resources, these methods have not been evaluated on large, complex datasets like XD-Violence, which involve more diverse and challenging scenarios. Among the seven weakly supervised methods, only Fan et al. [14] and RelVid achieved an AP surpassing 80%. Close behind are Zhang et al. [16], with an AP of 78.74%, and AVVD, with an AP of 78.10%. These results also reinforce our previous discussion namely, incorporating multimodal information inevitably introduces the risk of noise. The XD-Violence dataset, with its more diverse sources, presents greater challenges for anomaly detection. However, the inclusion of text guidance, to some extent, provides a form of constraint, drawing upon knowledge previously learned to refine the detection of anomalies. This illustrates the complex trade-off between leveraging additional modalities and managing the noise they may introduce.

In summary, RelVid introduces new methods that improve the performance of video anomaly recognition and detection, making it a promising approach for anomaly detection tasks in video data.

**Fine-grained WVAD results:** For the fine-grained WVAD task, RelVid is compared with previous works, AVVD and Sultani et al. [1,15], in Table 3 and Table 4.

RelVid achieves the best performance on the UCF-Crime dataset, demonstrating substantial improvements over Sultani et al. [1] and AVVD At an IoU of 0.1, RelVid’s mAP@IoU is 13.68%, surpassing AVVD by more than 4 percentage points. At higher IoU thresholds, RelVid continues to outperform other methods, reaching an average mAP@IoU of 6.83%, which is significantly higher than AVVD and Sultani et al. [1]

RelVid also excels on the XD-Violence dataset, achieving the highest mAP@IoU values at all thresholds, with a significant improvement over Sultani et al. [1], AVVD, and the Random Baseline. Specifically, RelVid reaches a mAP@IoU percentage of 42.30% at 0.1, much higher than the next best method, AVVD, which achieves 30.51%. Furthermore, the gap in the average mAP@IoU indicates RelVid’s superior overall performance, with an average of 28.33%, while other methods remain around 20%.

In summary, these results highlight that RelVid demonstrates superior performance across both the XD-Violence and UCF-Crime datasets, establishing it as a highly effective method for weakly supervised learning tasks.

### 4.5. Ablation Study

An ablation study was conducted to evaluate the contribution of individual components to model performance, as summarized in Table 5. In this study, Auxiliary Task 2 utilizes text information to assist the model in completing the VAR task. Feature activation (FA) and local attention serve different roles within the model, with FA enhancing feature representation and Local Attention improving contextual focus. The combination of these two components strengthens the model’s capability beyond what each module can achieve individually. However, this indicates the need for additional operations to balance the contributions of both components. To address this, we progressively introduce Auxiliary Task 1 and Auxiliary Task 2. Comparing the results, we find that Auxiliary Task 1 effectively balances the effects of FA and Local Attention, achieving a performance score of 87.30%. Ultimately, combining all components yields the best performance, further demonstrating the importance of their synergistic integration.

### 4.6. Discussion

**Cross-database quantitative results.** Since the types of anomalies present in both datasets are similar, the generalization ability of video anomaly detectors is crucial for real-world applications. This is because operations in different environments can vary significantly from the training and testing data. To evaluate the model’s performance across different data domains, experiments were conducted to assess its generalization ability, as shown in Table 6. These experiments demonstrate the model’s adaptability to domain differences by training on the source dataset and evaluating its performance on the target dataset. Specifically, while both the UCF-Crimes and XD-Violence datasets share similar definitions of abnormal events, XD-Violence is a larger dataset with a broader range of video types, including sports, surveillance, and movies. As a result, when XD-Violence is used as the source and UCF-Crimes as the target, the performance drop is minimal. In contrast, the reverse scenario is more representative of real-world environments. In this case, the proposed method shows a smaller performance drop compared to RTFM. This result highlights that RelVid is better equipped to handle domain differences than methods that focus solely on temporal dependencies, such as RTFM.

**Figure 5:**
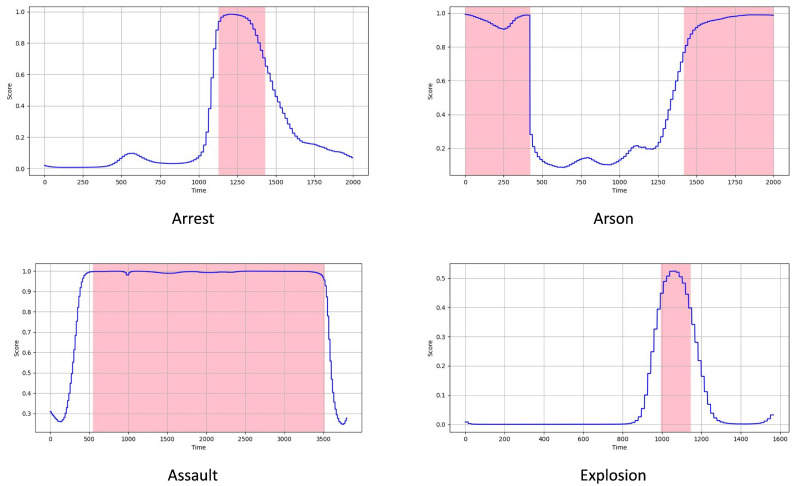
Examples of qualitative results on the UCF-Crime dataset.

**Qualitative Visualization.** The qualitative visualizations of the coarse-grained WSVAD are presented in Figure 5, where the blue curves represent the anomaly predictions and the pink regions indicate the ground-truth abnormal temporal locations. In Figure 5, the anomaly scores are normalized to the range of [0, 1] for easier comparison, as the score distributions vary for each method. From the plot, it is clear that the proposed method achieves more accurate temporal localization. As shown, RelVid successfully detects abnormal regions across different categories with high precision.

**Figure 6:**
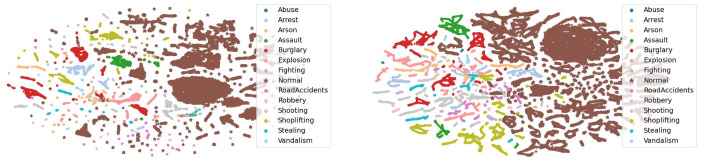
Visualization of final features on UCF-Crimes. The brown stars represent normal data, while the other colors indicate different types of anomalies. Compared to the left graph, the brown points (normal data) show fewer outliers. The features of other anomaly categories are also better clustered.

**Feature visualization.** The features of the UCF-Crime dataset were visualized using t-SNE, as shown in Figure 6. The results reveal that, while the CLIP model demonstrates strong capability in capturing general features through the learning of image-text pairs, it struggles to effectively differentiate between WVAD categories due to the inherent complexities of the task. In contrast, after optimization with the RelVid model, the visual feature distribution exhibits more distinct category boundaries. Notably, the features of the normal category are clustered in the upper-left corner, with no scattered abnormal features, indicating that the RelVid model is more effective at distinguishing between normal and abnormal categories.

## 5. Conclusions

This paper introduces RelVid, a novel paradigm for weakly supervised video anomaly detection (WVAD) that leverages pre-trained knowledge and visual-language associations from a frozen CLIP model. The framework incorporates two auxiliary tasks: reconstruction and text-guided anomaly recognition. To capture more comprehensive class-specific temporal information, an Adapter is proposed alongside a feature activation (FA) mechanism designed to selectively activate class-specific features. Furthermore, relative distance learning is employed to enhance class separation, while the introduction of local attention improves the model’s ability to capture rich contextual information.

Compared to existing semi-supervised models, RelVid effectively addresses the challenge of handling large-scale, complex datasets. Traditional semi-supervised models often struggle with such datasets due to their resource-intensive training processes and their focus on small-scale data for training and evaluation. An extensive comparison with state-of-the-art semi-supervised methods reveals that these methods consistently lag significantly behind the WVAD method in both accuracy and the computational resources required for training.

Experimental results show that WVAD significantly improves model performance with a limited number of video-level labels. By incorporating text-guided information, RelVid provides an additional basis for classification, greatly enhancing feature refinement and enabling the model to more effectively distinguish anomalous events. RelVid performs well, particularly on the UCF-Crime dataset, but faces challenges when applied to the more complex XD-Violence dataset. This highlights that while integrating multimodal information is beneficial, further refinement is necessary to prevent the introduced information from interfering with the original image features. Without such refinement, excessive information may increase noise, ultimately hindering the model’s ability to capture meaningful features and temporal dependencies effectively.

Future work will focus on applying visual-linguistic pre-training to open-set VAD tasks and exploring its use in real-world scenarios with diverse and difficult-to-define anomalies. Additionally, the research will address how to preserve image recognition capabilities when integrating other modal information, such as text, to ensure the model remains robust in anomaly detection.

## Figures and Tables

**Table 1:** Comparison results with other methods in UCF-Crime datasets.

Category	Methods	VAD	VAR	AUC (%)	Ano-AUC (%)
Semi	SVM baseline	✓	×	50.10	50.00
	OCSVM	✓	×	63.20	51.06
	Hasan et al. [19]	✓	×	51.20	39.43
	BODS	✓	×	68.26	N/A
	GODS	✓	×	70.46	N/A
	S3R	✓	×	79.58	N/A
	GCL	✓	×	71.04	N/A
Weak	Sultani et al. [1]	✓	×	84.14	63.29
	RTFM	✓	×	85.66	63.86
	AVVD	✓	×	82.45	60.27
	Ju et al. [9]	✓	×	84.72	62.60
	UMIL	✓	×	86.75	68.68
	Zhang et al. [16]	✓	×	86.22	N/A
	Fan et al. [14]	✓	×	86.19	N/A
	RelVid (Ours)	✓	✓	**87.71**	**69.29**

The bold text indicates the highest score.

**Table 2:** Comparison results with other methods on the XD-Violence dataset.

Category	Methods	VAD	VAR	AP (%)
Semi	SVM baseline	✓	×	50.80
	Hasan et al. [19]	✓	×	28.63
	OCSVM	✓	×	31.25
	S3R	✓	×	53.52
Weak	Sultani et al. [1]	✓	×	75.18
	RTFM	✓	×	77.81
	AVVD	✓	×	78.64
	Ju et al. [9]	✓	×	76.57
	Fan et al. [14]	✓	×	**83.59**
	Zhang et al. [16]	✓	×	78.74
	RelVid (Ours)	✓	✓	80.76

The bold text indicates the highest score.

**Table 3:** Comparison of mAP@IOU results on the UCF-Crime dataset.

Method	mAP@IOU (%)					
	0.1	0.2	0.3	0.4	0.5	AVG
Random Baseline	0.21	0.14	0.04	0.02	0.01	0.08
Sultani et al. [1]	5.73	4.41	2.69	1.93	1.44	3.24
AVVD	10.27	7.01	6.25	3.42	3.29	6.05
RelVid (Ours)	13.68	7.34	5.39	4.12	3.61	6.83

**Table 4:** Comparison of mAP@IOU results on the XD-Violence dataset.

Method	mAP@IOU (%)					
	0.1	0.2	0.3	0.4	0.5	AVG
Random Baseline	1.82	0.92	0.48	0.23	0.09	0.71
Sultani et al. [1]	22.72	15.57	9.98	6.20	3.78	11.65
AVVD	30.51	25.75	20.18	14.83	9.79	20.21
RelVid	42.30	34.66	28.04	20.94	15.70	28.33

**Table 5:** Comparison of different components.

FA	Local Attention	Auxiliary Task 1	Auxiliary Task 2	AUC (%)
✓				84.64
	✓			86.37
✓	✓			85.73
	✓	✓		86.86
	✓	✓	✓	87.13
✓	✓	✓		87.30
✓	✓	✓	✓	**87.71**

**Table 6:** Cross-database experimental results on the UCF-Crimes and XD-Violence datasets.

Source	UCF-Crimes	XD-Violence	XD-Violence	UCF-Crimes
Target	UCF-Crimes		XD-Violence	
RTFM	84.48	68.59 (18.80% ↓)	76.62	37.30 (39.32% ↓)
Ours	87.71	84.61 (3.1% ↓)	80.76	62.87 (17.89% ↓)

## Data Availability

The datasets are publicly available at https://www.crcv.ucf.edu/projects/real-world/, (accessed on 20 February 2025) and https://roc-ng.github.io/XD-Violence/ (accessed on 20 February 2025).

## References

[1] Sultani W., Chen C., Shah M. Real-world anomaly detection in surveillance videos. Proceedings of the IEEE Conference on Computer Vision and Pattern Recognition.

[2] Tran D., Bourdev L., Fergus R., Torresani L., Paluri M. Learning spatiotemporal features with 3d convolutional networks. Proceedings of the IEEE International Conference on Computer Vision.

[3] Zaheer M.Z., Mahmood A., Astrid M., Lee S.I. (2020). Claws: Clustering assisted weakly supervised learning with normalcy suppression for anomalous event detection. Proceedings of the Computer Vision–ECCV 2020: 16th European Conference.

[4] Carreira J., Zisserman A. Quo vadis, action recognition? a new model and the kinetics dataset. Proceedings of the IEEE Conference on Computer Vision and Pattern Recognition.

[5] Wu P., Liu J., Shi Y., Sun Y., Shao F., Wu Z., Yang Z. (2020). Not only look, but also listen: Learning multimodal violence detection under weak supervision. Proceedings of the Computer Vision–ECCV 2020: 16th European Conference.

[6] Wu J.C., Hsieh H.Y., Chen D.J., Fuh C.S., Liu T.L. (2022). Self-supervised sparse representation for video anomaly detection. Proceedings of the European Conference on Computer Vision.

[7] Zhou Y., Qu Y., Xu X., Shen F., Song J., Shen H.T. (2024). Batchnorm-based weakly supervised video anomaly detection. IEEE Trans. Circuits Syst. Video Technol..

[8] AlMarri S., Zaheer M.Z., Nandakumar K. A Multi-Head Approach with Shuffled Segments for Weakly-Supervised Video Anomaly Detection. Proceedings of the IEEE/CVF Winter Conference on Applications of Computer Vision.

[9] Ju C., Han T., Zheng K., Zhang Y., Xie W. (2022). Prompting visual-language models for efficient video understanding. Proceedings of the European Conference on Computer Vision.

[10] Wan B., Fang Y., Xia X., Mei J. Weakly supervised video anomaly detection via center-guided discriminative learning. Proceedings of the 2020 IEEE International Conference on Multimedia and Expo (ICME).

[11] Zhong J.X., Li N., Kong W., Liu S., Li T.H., Li G. Graph convolutional label noise cleaner: Train a plug-and-play action classifier for anomaly detection. Proceedings of the IEEE/CVF Conference on Computer Vision and Pattern Recognition.

[12] Tian Y., Pang G., Chen Y., Singh R., Verjans J.W., Carneiro G. Weakly-supervised video anomaly detection with robust temporal feature magnitude learning. Proceedings of the IEEE/CVF International Conference on Computer Vision.

[13] Lv H., Yue Z., Sun Q., Luo B., Cui Z., Zhang H. Unbiased multiple instance learning for weakly supervised video anomaly detection. Proceedings of the IEEE/CVF Conference on Computer Vision and Pattern Recognition.

[14] Fan Y., Yu Y., Lu W., Han Y. (2024). Weakly-supervised video anomaly detection with snippet anomalous attention. IEEE Trans. Circuits Syst. Video Technol..

[15] Wu P., Liu X., Liu J. (2022). Weakly supervised audio-visual violence detection. IEEE Trans. Multimed..

[16] Zhang C., Li G., Qi Y., Wang S., Qing L., Huang Q., Yang M.H. Exploiting completeness and uncertainty of pseudo labels for weakly supervised video anomaly detection. Proceedings of the IEEE/CVF Conference on Computer Vision and Pattern Recognition.

[17] Feng J.C., Hong F.T., Zheng W.S. Mist: Multiple instance self-training framework for video anomaly detection. Proceedings of the IEEE/CVF Conference on Computer Vision and Pattern Recognition.

[18] Schölkopf B., Williamson R.C., Smola A., Shawe-Taylor J., Platt J. Support vector method for novelty detection. Proceedings of the 13th International Conference on Neural Information Processing Systems.

[19] Hasan M., Choi J., Neumann J., Roy-Chowdhury A.K., Davis L.S. Learning temporal regularity in video sequences. Proceedings of the IEEE Conference on Computer Vision and Pattern Recognition.

[20] Wang J., Cherian A. Gods: Generalized one-class discriminative subspaces for anomaly detection. Proceedings of the IEEE/CVF International Conference on Computer Vision.

[21] Zaheer M.Z., Mahmood A., Khan M.H., Segu M., Yu F., Lee S.I. Generative cooperative learning for unsupervised video anomaly detection. Proceedings of the IEEE/CVF Conference on Computer Vision and Pattern Recognition.

[22] Zhang J., Qing L., Miao J. Temporal convolutional network with complementary inner bag loss for weakly supervised anomaly detection. Proceedings of the 2019 IEEE International Conference on Image Processing (ICIP).

[23] Wu P., Liu J. (2021). Learning causal temporal relation and feature discrimination for anomaly detection. IEEE Trans. Image Process..

[24] Chen D., Wang P., Yue L., Zhang Y., Jia T. (2020). Anomaly detection in surveillance video based on bidirectional prediction. Image Vis. Comput..

[25] Ganokratanaa T., Aramvith S., Sebe N. (2020). Unsupervised anomaly detection and localization based on deep spatiotemporal translation network. IEEE Access.

[26] Park C., Cho M., Lee M., Lee S. FastAno: Fast anomaly detection via spatio-temporal patch transformation. Proceedings of the IEEE/CVF Winter Conference on Applications of Computer Vision.

[27] Dosovitskiy A. (2020). An image is worth 16 × 16 words: Transformers for image recognition at scale. arXiv.

[28] Radford A., Kim J.W., Hallacy C., Ramesh A., Goh G., Agarwal S., Sastry G., Askell A., Mishkin P., Clark J. Learning transferable visual models from natural language supervision. Proceedings of the International Conference on Machine Learning.

[29] Zhou X., Girdhar R., Joulin A., Krähenbühl P., Misra I. Detecting twenty-thousand classes using image-level supervision. Proceedings of the European Conference on Computer Vision.

[30] Xu H., Ghosh G., Huang P.Y., Okhonko D., Aghajanyan A., Metze F., Zettlemoyer L., Feichtenhofer C. (2021). Videoclip: Contrastive pre-training for zero-shot video-text understanding. arXiv.

[31] Zanella L., Liberatori B., Menapace W., Poiesi F., Wang Y., Ricci E. (2024). Delving into clip latent space for video anomaly recognition. Comput. Vis. Image Underst..

[32] Wu X., He R., Sun Z., Tan T. (2018). A light CNN for deep face representation with noisy labels. IEEE Trans. Inf. Forensics Secur..

[33] Zhou K., Yang J., Loy C.C., Liu Z. (2022). Learning to prompt for vision-language models. Int. J. Comput. Vis..

